# EFFECT OF EARLY HYBRID ASSISTIVE LIMB ASSISTED REHABILITATION ON FUNCTIONAL OUTCOMES AND PATIENT SATISFACTION AFTER TOTAL KNEE ARTHROPLASTY: A RANDOMIZED CONTROLLED TRIAL

**DOI:** 10.2340/jrm.v58.43925

**Published:** 2026-03-09

**Authors:** Takaya WATABE, Ryota MURAMATSU, Takuya SENGOKU, Goro SAKURAI, Shinya YOSHIDA, Yuta TANIGUCHI

**Affiliations:** 1 Section of Rehabilitation, Kanazawa University Hospital, Kanazawa, Ishikawa; 2 Division of Mechanical Science and Engineering Graduate School of Natural Science and Technology, Kanazawa University, Kakuma-Machi, Kanazawa, Ishikawa; 3 Department of Orthopedic Surgery, Graduate School of Medicine Sciences, Kanazawa University, Takara-Machi, Kanazawa, Ishikawa, Japan

**Keywords:** knee osteoarthritis, early rehabilitation, robot rehabilitation, total knee arthroplasty

## Abstract

**Background:**

Early rehabilitation within 4 h following total knee arthroplasty involves passive exercise with manual therapy.

**Objective:**

The aim was to determine the beneficial effects of the single-joint hybrid assistive limb for rehabilitation within 4 h after total knee arthroplasty.

**Methods:**

This single-blinded randomized controlled trial included 68 participants who underwent primary total knee arthroplasty for knee osteoarthritis and were assigned to the early single-joint hybrid assistive limb (*n* = 22), HAL-SJ (*n* = 23), and control (*n* = 23) groups.

**Results:**

The Knee Injury and Osteoarthritis Outcome Score quality of life (*p* = 0.007) scores of the early single-joint hybrid assistive limb group showed significantly higher values than the control groups at 12 months. The recovery time for extension lag was significantly improved in the early single-joint hybrid assistive limb group compared with the single-joint hybrid assistive limb (*p* = 0.006) and control (*p* < 0.001) groups. Additionally, the knee flexion range of motion of the early single-joint hybrid assistive limb group showed significantly higher values than the single-joint hybrid assistive limb (*p* = 0.029) and control (*p* = 0.031) groups at 1 week.

**Conclusions:**

Early single-joint hybrid assistive limb rehabilitation may improve patient-reported quality of life at 12 months without exacerbating postoperative pain or swelling. These results suggest that the single-joint hybrid assistive limb may be a feasible adjunct to early postoperative rehabilitation after total knee arthroplasty, while further studies are required to clarify its clinical relevance and long-term benefits.

## LAY ABSTRACT

This study investigated whether using a wearable robotic device called the single-joint hybrid assistive limb can improve early rehabilitation after total knee arthroplasty. A total of 68 patients were divided into 3 groups, with 1 group starting hybrid assistive limb therapy within 4 h after surgery. The early hybrid assistive limb group showed better quality of life 12 months postoperatively compared with the other groups. They also recovered knee extension and range of motion more quickly. These results suggest that starting with the hybrid assistive limb soon after surgery may lead to faster and better recovery in patients undergoing total knee arthroplasty. These results suggest that HAL-SJ may be a feasible adjunct to early postoperative rehabilitation after total knee arthroplasty, while further studies are required to clarify its clinical relevance and long-term benefits.

Osteoarthritis (OA) is a common age-related disease that causes pain, stiffness, and loss of knee function (1). Total knee arthroplasty (TKA) is one of the most common surgical procedures for patients with end-stage knee OA (2). Owing to the rising prevalence of OA worldwide, the frequency of TKA is also increasing, resulting in a significant burden on medical expenses (3). Early rehabilitation within 24 h following TKA is associated with a shorter hospital stay, lower overall costs, and reduced risk of complications such as deep vein thrombosis and cystitis (4-7). Early rehabilitation within 4 h of TKA improved early extension range of motion (ROM), reduced pain, and improved the gait pattern compared with standard rehabilitation (8). Additionally, knee flexion ROM and an improved gait pattern contribute to better functional outcomes at 1 year postoperatively (9). However, although outcomes after TKA generally improve, up to 20% of patients experience prolonged pain, joint stiffness, and reduced satisfaction (10, 11). Therefore, new rehabilitation strategies are important to improve knee joint function and patient satisfaction.

In recent years, the single-joint hybrid assistive limb (HAL-SJ; Cyberdyne Inc, Tsukuba, Japan) has attracted attention as a new rehabilitation tool in musculoskeletal rehabilitation for use after TKA (12, 13). The length of hospital stay in the robot-assisted rehabilitation group is shorter than that in the traditional therapy group, and the total cost of hospitalization was lower (14). Unlike continuous passive motion devices, which provide passive motion without patient involvement, the HAL-SJ facilitates active-assistive movement by detecting bioelectrical signals (BES) from muscles, promoting voluntary muscle activation and neuromuscular control. Previous studies that conducted knee joint extension training using the HAL-SJ after TKA compared with standard rehabilitation reported improved extension lag, gait function, and reduced knee pain (12, 13, 15–19). In addition, patient satisfaction scores with patient-reported outcomes were significantly improved in the HAL-SJ group (12, 17, 20). However, while short-term results have been positive, the long-term effectiveness of rehabilitation with the HAL-SJ remains unclear.

This single-blinded randomized controlled trial aimed to evaluate the efficacy of early knee exercise within 4 h after TKA using the HAL-SJ. Specifically, we evaluated the efficacy of early knee exercise using the HAL-SJ by comparing longitudinal changes in the Knee Injury and Osteoarthritis Outcome Score (KOOS) between the early HAL-SJ group and the usual care group over the postoperative follow-up period. As a secondary objective, outcomes in the HAL-SJ group were examined to explore the potential influence of intervention timing. We hypothesized that early initiation of knee exercises using the HAL-SJ would enhance early knee joint function (the time it takes for recovery extension lag and knee ROM) and improve patient-reported outcomes compared with conventional physical therapy without the HAL-SJ.

## METHODS

### Study design

This single-blinded randomized controlled trial conforms to the Consolidated Standards of Reporting Trials 2010 statement (21). Eligible participants underwent a baseline assessment conducted by a blinded assessor and were randomized into 1 of the 3 intervention groups. Blocked, stratified randomization was performed by a physiotherapist using a web-based server (UMIN Internet Data and Information System for Clinical and Epidemiological Research, Japan) with a random list pre-generated by the software. All patients provided written informed consent to participate in the study. This study was conducted in accordance with the Declaration of Helsinki and was approved by our institution’s Ethics Committee (No. 113786). This study was registered with the University Hospital Medical Information Network (UMIN000053675).

### Participants

Eighty Japanese patients with end-stage OA who underwent primary TKA using the Robotic Surgical Assistant Knee System (Zimmer Biomet, Warsaw, IN, USA) and the subvastus approach at a single university hospital between October 2021 and August 2023 were recruited for this study. All operations were performed by 2 arthroplasty surgeons with 10 years of experience in the field. Patients with rheumatoid arthritis, severe cognitive deficits that interfere with activities of daily living or decision-making or mental disorders, Kellgren–Lawrence score < 2, paralysis of the lower extremity, body mass index > 35 kg/m^2^, and pain that made conventional rehabilitation impossible; those with a history of previous tibial osteotomy, revision arthroplasty, and non-device related revision surgery; and those who declined to participate in the study were excluded. Five patients who met at least 1 of these criteria were excluded. Therefore, 75 patients were included and assigned to 1 of 3 groups: early HAL-SJ (HAL-SJ exercises within 4 h of TKA), HAL-SJ (HAL-SJ exercises commenced 7 days after TKA), or the control group.

### Measurements

*Primary outcome.* The primary outcome was the KOOS, which was used to evaluate functional and patient-reported outcomes preoperatively and at 12 months postoperatively. The KOOS is a validated score for TKA that can be used to evaluate long-term effects and physical therapy (22). The subscales range from 0 to 100, with 0 representing the worst possible score and 100 the best possible score (23).

*Secondary outcomes.* The secondary outcomes included passive ROM, measured preoperatively and at 1 and 2 weeks, 3, 6, and 12 months postoperatively. Other secondary outcomes measured were the recovery time for extension lag, quadriceps and hamstring isometric knee strength, knee pain intensity numerical rating scale (NRS) scores, walking speed, and knee circumference at 2 weeks postoperatively. Passive ROM was measured in 5 increments using a goniometer (Toudaisiki-Goniometer, OG Wellness Co Ltd, Okayama, Japan). Extension lag, defined as the difference between the active and passive ranges of knee extension, was measured using a goniometer by licensed physical therapists. Quadriceps and hamstring strength were measured using a handheld dynamometer (μTas F-1, ANIMA, Tokyo, Japan). Patient hips and knees were positioned at 90°. The length of the straps allowed for an isometric contraction to be performed with the knee at 90° during both flexion and extension (24). Knee circumference was measured with the participants relaxed in the supine position and the knees extended. Measurements were obtained 1 cm proximal to the upper edge of the patella using a non-stretchable tape measure. Two measurements were acquired, and the mean was used for analysis. Previous studies have demonstrated excellent intra-rater and good inter-rater reliabilities for circumference measurements using a tape measure (25). The 10 m walking speed was measured at a comfortable pace along a 14 m straight walking path, including a 2 m front and rear runway. The time required for patients to walk 10 m was measured using a stopwatch (26).

### Interventions and rehabilitation protocol

**Fig. 1:**
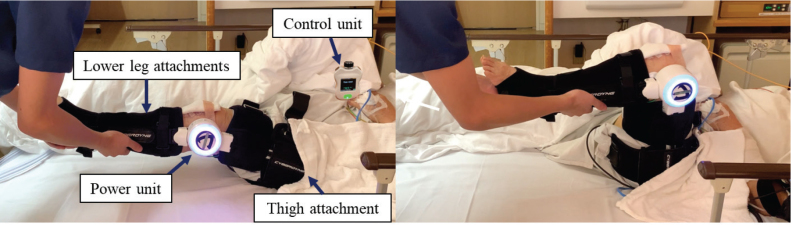
Early initiation of exercises in the early HAL-SJ group. Overview of single-joint Hybrid Assistive Limb (HAL-SJ). Patients in the early HAL-SJ group perform extension and flexion exercises using the affected knee joint while lying in bed within 4 h after surgery.

During hospitalization, all patients participated in a 40-min rehabilitation session each day, 5 times a week. The rehabilitation protocol was consistent across all patients. The timing of HAL-SJ intervention was determined based on the study group: the early HAL-SJ group began exercising with HAL-SJ within the first 4 h after surgery, while the HAL-SJ group started HAL-SJ exercises on postoperative day 7. On the day of surgery (day 0), patients in the HAL-SJ and control groups remained in bed without rehabilitation. The early HAL-SJ group commenced rehabilitation within 4 h after surgery, primarily focusing on active-assisted mobilization using the HAL-SJ (Fig. 1).

**Fig. 2:**
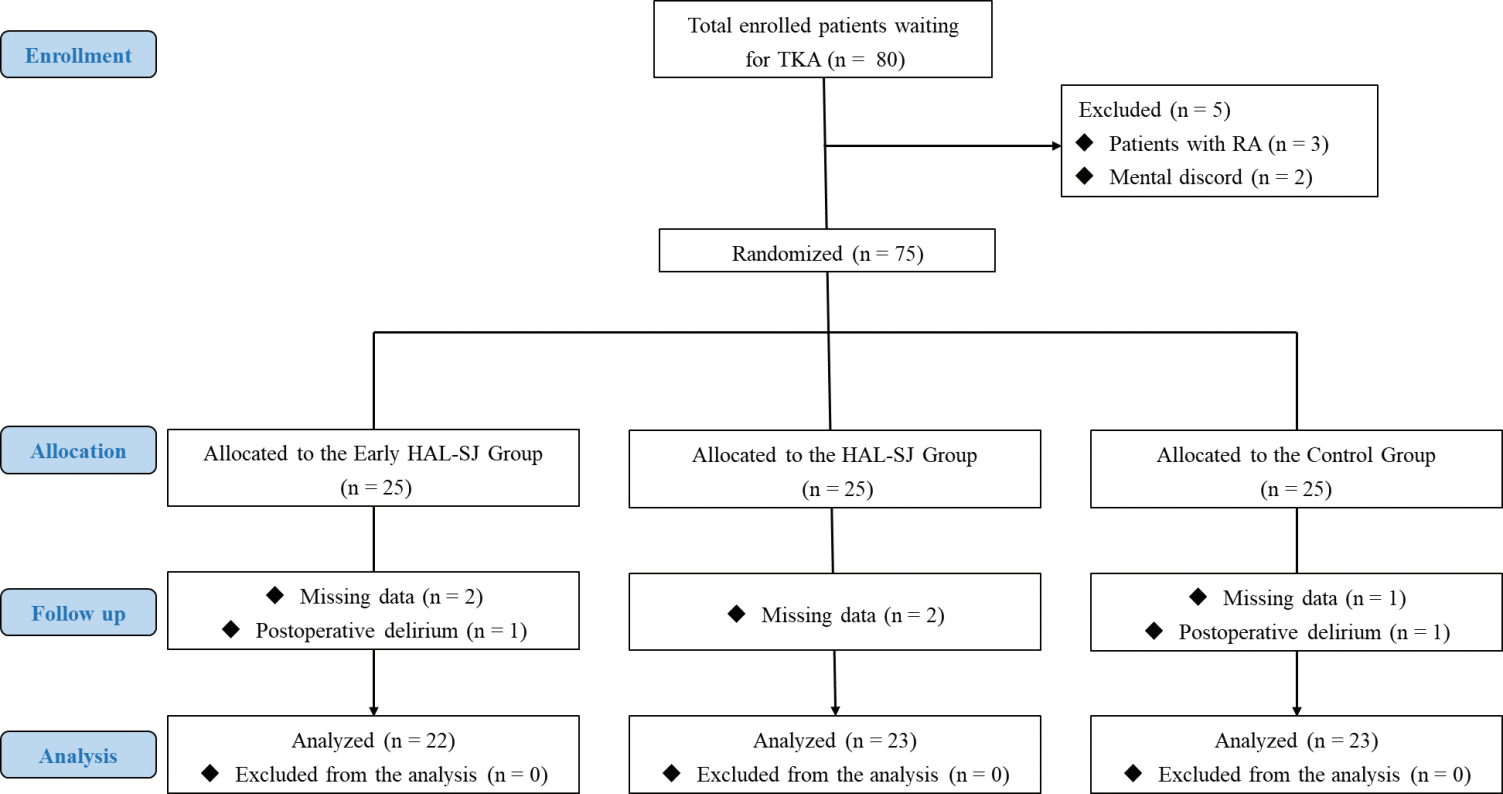
Flow diagram of patient selection for the study. TKA: total knee arthroplasty; RA: rheumatoid arthritis; HAL-SJ: single-joint hybrid assistive limb.

On day 1, patients in all groups initiated exercises, including sitting in bed, transferring to wheelchairs, standing, walking short distances on flat ground, flexion–extension exercises, and isotonic muscle contractions while seated. From day 2 until discharge, the regimen included active-resisted quadriceps exercises, progressively intensive exercises with walking aids, increased walking distances, gait re-education, and adaptation to daily activities. The early HAL-SJ group resumed HAL-SJ exercises from day 7 onwards, continuing the postoperative exercises that began 4 h after surgery. Meanwhile, the HAL-SJ group started using the HAL-SJ on postoperative day 7. Patients in both groups performed active-assisted mobilization using the HAL-SJ, involving the extension and flexion of the affected knee joint while seated in bed (Fig. 2).

The BES of the vastus medialis and biceps femoris muscles were recorded using electrodes placed according to the Surface Electromyography for the Non-Invasive Assessment of Muscles guidelines. In cybernic voluntary-control mode (CVC-AutoEXT/AutoFlx), patients received assistance from BES triggered by knee joint extension and flexion movements. The settings were adjusted to an extension/flexion signal balance with flexion at 80% and extension at 100%, assist gain between 20% and 40%, and a torque limit of 50%. Initially, knee joint flexion started at 90°, increasing as the patient’s capacity improved, reaching up to 120°. Five sets of 10 repetitions were performed 3 times a week (with a 1-min rest between sets, totalling 15–20 min per session) (12, 17). All rehabilitation programmes were managed by a physical therapist, carefully monitoring for worsening pain. Both groups also received routine treatments to prevent deep vein thrombosis, including calf muscle air pumping and active ankle dorsiflexion–plantar flexion exercises. Postoperative pain management was identical in both groups, involving an adductor canal block with 20 mL of ropivacaine (7.5 mg/mL) administered by the anaesthesiologist during surgery. Intravenous opioid analgesics were given as needed on the first postoperative day, followed by oral celecoxib at 200 mg/day for 1 week starting the next day.

### Statistical analysis

G* Power 3.1.9.6 (Franz Paul, Kiel, Germany) was used to determine the sample size for this study, based on an anticipated minimum clinically important difference of a 16% increase in the KOOS at 1 year postoperatively and an assumed standard deviation of 20% (27), with an effect size of 0.40 for a 3-group comparison. Therefore, 75 patients were eligible and followed up. A generalized linear mixed-effects model with both random intercepts and slopes (lme4 package of R; R Foundation for Statistical Computing, Vienna, Austria) was applied to compare the KOOS score, quadriceps and hamstring strength, walking speed, resting and walking pain scores, and knee flexion and extension ROM. Covariates included group (early HAL-SJ, HAL-SJ and control) and time (pre and 2 weeks or 12 months). For knee flexion and extension ROM, additional time (pre, 1-week, 2 weeks, 3 months, 6 months, and 12 months) for knee flexion and extension ROM was included. A Bonferroni correction was applied to adjust the effects of multiple testing for multiple comparisons. As the KOOS consists of 5 subscales and each subscale was analysed using a separate statistical model, a Bonferroni correction was applied to control for multiplicity. Accordingly, statistical significance for the primary outcome was defined as *p* < 0.01 (0.05/5). Partial eta squared (η²) was used to calculate effect sizes, with thresholds set at 0.01 for small, 0.06 for medium, and 0.14 for large effects. All statistical analyses were performed using R (version 3.6.2), and the level of statistical significance was set at 0.05.

## RESULTS

Seventy-five patients met the inclusion/exclusion criteria and were recruited for this study. However, 7 patients (3 in the early HAL-SJ group, 3 in the HAL-SJ group, and 1 in the control group) withdrew because of (*i*) missing data and (*ii*) postoperative delirium. Therefore, 68 patients (22, 23, and 23 in the early HAL-SJ, HAL-SJ, and control groups, respectively) were considered for the final evaluation (Fig. 2, Table I).

**Table I:** Patient demographics data

Variable	Early HAL-SJ group (*n* = 22)	HAL-SJ group (*n* = 23)	Control group (*n* = 23)
Sex males:females, *n*	8:14	5:18	5:18
Age at time of surgery, years, mean (SD)	76.9 (5.2)	73.8 (6.1)	75.2 (6.5)
Affected side left:right, *n*	9:13	10:13	10:13
Height, cm, mean (SD)	156.0 (9.6)	155.7 (6.4)	155.0 (10.1)
Weight, kg, mean (SD)	62.1 (9.7)	62.5 (8.4)	61.1 (8.5)
BMI, kg/m^2^, mean (SD)	25.6 (4.0)	25.6 (2.4)	25.6 (4.1)
Kellgren–Lawrence grade, *n*	III: 5, IV: 17	III: 4, IV: 19	III: 5, IV: 18

SD: standard deviation; BMI: body mass index; HAL-SJ: single-joint hybrid assistive limb.

### Primary outcome

**Fig. 3:**
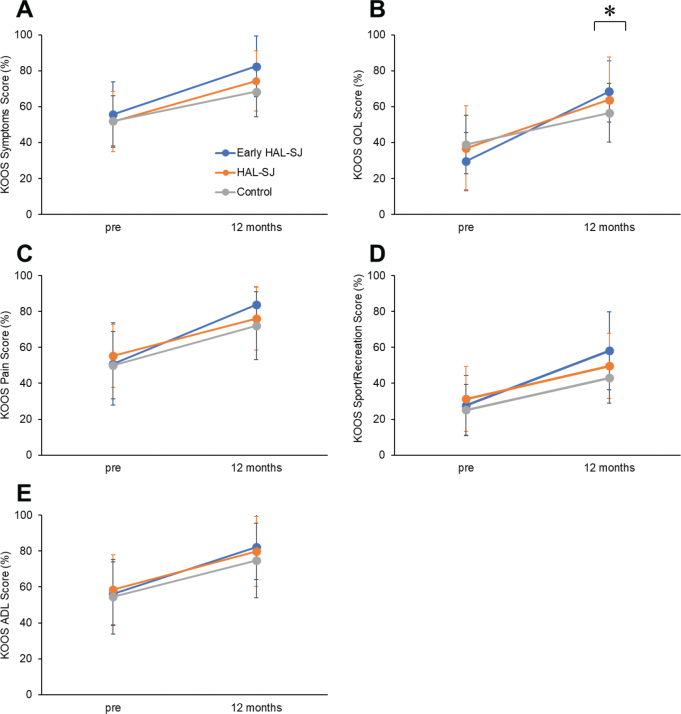
KOOS subscale scores: preoperatively to 12 months after TKA. Mean KOOS subscale scores with 95% CI. * Early HAL-SJ group vs Control group (*p* < 0.01). KOOS: Knee Injury and Osteoarthritis Outcome Score; TKA: total knee arthroplasty; HAL-SJ: single-joint hybrid assistive limb; ADL: activities of daily living; QOL: quality of life.

The primary outcome revealed significant interactions of the time × groups for the KOOS quality of life (QOL) (*p* < 0.001) (Table II). The KOOS QOL scores in the early HAL-SJ group were significantly higher values than those in the control groups at 12 months (early HAL-SJ: 95% CI = 60.14 to 74.10; control: 95% CI = 46.84 to 67.95; *p* = 0.007, see Fig. 3D). A significant time main effect was observed for the KOOS pain, activities of daily living (ADL), and sport/recreation scores (*p* < 0.001). The KOOS scales of all groups exhibited significantly higher values at 12 months compared with the preoperative scores (Fig. 3).

**Table II:** Clinical results of the groups

Clinical results	Groups, mean (SD)			*p*-value	ES (η^2^)	Post hoc, mean (SD)		
	Early HAL-SJ	HAL-SJ	Control			Early vs HAL	Early vs Control	HAL vs Control
Hospitalization duration (days)	18.0 (3.3)	17.4 (2.9)	17.7 (3.1)	0.833	0.004	–	–	–
Inpatient rehabilitation (days)	12.9 (2.9)	11.6 (1.5)	12.1 (2.5)	0.174	0.052	–	–	–
Using HAL-SJ (times)	7.7 (1.0)	7.5 (1.2)	0.0 (0.0)	NA	–	–	–	–
Extension lag (days)	7.6 (2.8)	11.1 (4.4)	13.1 (3.4)	**0.001**	0.289	**0.006**	**< 0.001**	0.135

SD: standard deviation; ES: effect size; HAL-SJ: single-joint hybrid assistive limb. Significant differences are shown in bold.

### Secondary outcomes

The recovery time for extension lag was significantly improved in the early HAL-SJ group compared with the HAL-SJ (*p* = 0.006, average differential value = 3.451, 95% confidence interval (CI) = 2.671 to 9.571) and control (*p* < 0.001, average differential value = 5.538, 95% CI = 2.668 to 13.748) groups. However, no significant differences were observed between the groups regarding hospitalization duration and total days of inpatient rehabilitation (excluding weekends) (Table II). Significant time main effects were observed for hamstring strength, with significantly decreased hamstring strength at 2 weeks compared with preoperative values (*p* = 0.002, η^2^ = 0.079) (Table III). Significant time main effects were observed for resting pain, with significantly decreased resting pain NRS scores at 2 weeks compared with preoperative values (*p* < 0.001, η^2^ = 0.509). In addition, significant time main effects were observed for knee circumference of 1 cm, with significantly increased knee circumference of 1 cm at 2 weeks compared with preoperative values (*p* < 0.001, η^2^ = 0.155). No significant differences were observed in the quadriceps isometric knee strength, walking speed, and walking NRS pain score.

**Table III:** Pre- and postoperative secondary outcomes of the groups

Clinical results	Time	Groups, mean (SD)			*p*-value (effect size, η^2^), mean (SD)		
		Early HAL-SJ	HAL-SJ	Control	Time × Group	Time	Group
Quadriceps strength (N/kg)	Pre	3.2 (1.4)	3.0 (0.8)	3.1 (1.2)	0.994 (0.000)	0.870 (0.000)	0.271(0.022)
	2 weeks	2.4 (1.0)	2.4 (0.8)	2.2 (0.7)			
Hamstring strength (N/kg)	Pre	1.6 (0.5)	1.8 (0.5)	1.5 (0.6)	0.719 (0.005)	**0.002 (0.079)**	0.267 (0.022)
	2 weeks	1.2 (0.5)	1.3 (0.4)	1.1 (0.4)			
Walking speed (m/s)	Pre	0.9 (0.2)	0.8 (0.2)	0.8 (0.2)	0.109 (0.035)	0.089 (0.023)	0.766 (0.004)
	2 weeks	0.9 (0.2)	0.8 (0.2)	0.8 (0.2)			
Resting pain NRS	Pre	3.6 (1.2)	4.4 (1.4)	3.6 (1.6)	0.291 (0.019)	**< 0.001 (0.509)**	0.193 (0.025)
	2 weeks	1.0 (1.4)	1.2 (1.3)	1.2 (1.3)			
Walking pain NRS	Pre	4.2 (1.1)	4.6 (1.1)	4.2 (1.6)	0.177 (0.026)	0.081 (0.023)	0.361 (0.016)
	2 weeks	2.1 (1.4)	2.4 (1.4)	3.0 (1.4)			
Knee circumference (cm)	Pre	39.4 (1.6)	39.0 (2.4)	39.0 (1.7)	0.590 (0.009)	**< 0.001 (0.155)**	0.861 (0.003)
	2 weeks	40.8 (2.1)	41.3 (3.3)	41.6 (2.6)			

SD: standard deviation; NRS: numeric rating scale; HAL-SJ: single-joint hybrid assistive limb.

**Fig. 4:**
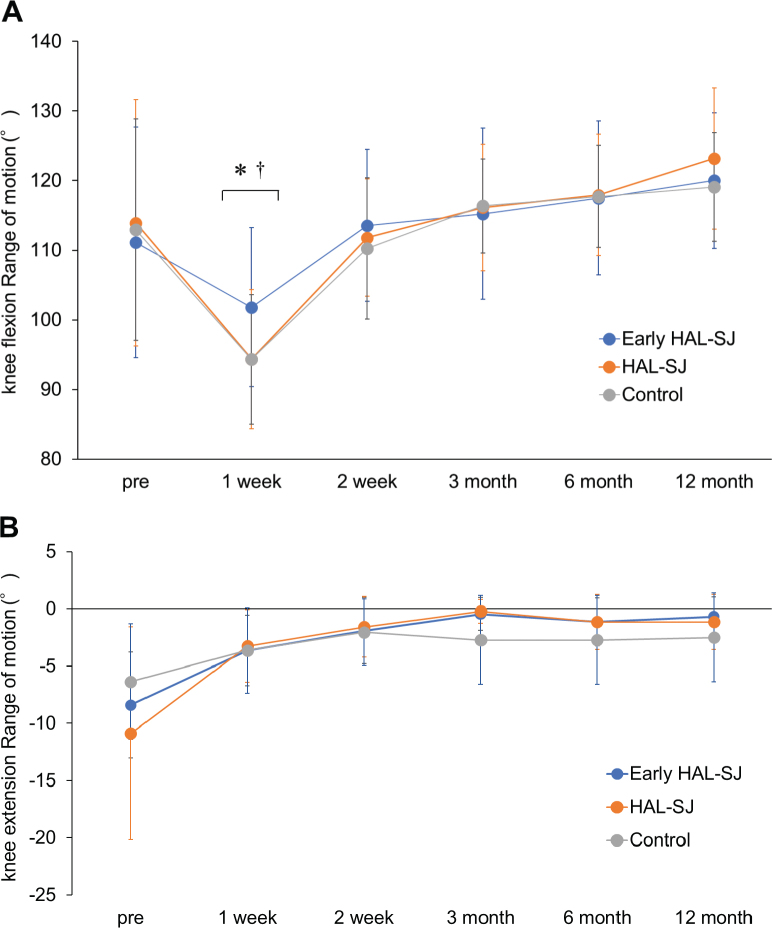
Comparison of the knee flexion and extension ranges of motion among all the groups. (A) Mean knee flexion range of motion over time with 95% CI. (B) Mean knee extension range of motion over time with 95% CI. * Early HAL-SJ group vs HAL group, † Early HAL-SJ group vs Control group (*p* < 0.05). HAL-SJ: single-joint hybrid assistive limb; CI: confidence interval.

The knee flexion ROM in the early HAL-SJ group was significantly higher than those in HAL-SJ (early HAL-SJ; 95% CI = 97.40 to 106.95°; HAL-SJ; 95% CI = 90.21 to 98.22°; *p* = 0.029) and control (early HAL-SJ; 95% CI = 97.40 to 106.95°; control; 95% CI = 90.72 to 98.41°; *p* = 0.031) groups at 1 week after TKA (Fig. 4A). A significant time main effect was observed for the knee extension ROM (*p* < 0.001, η^2^ = 0.184). The knee extension ROM exhibited significantly higher values from 2 weeks to 12 months compared with 1 week after TKA (*p* < 0.001) (Fig. 4B). No immediate adverse events, such as increased pain, bleeding, or other complications, were observed following the initial intervention in the early HAL-SJ group. Furthermore, no intervention-related adverse events were reported during the follow-up period up to 1 year postoperatively, supporting the safety of this approach not only in the acute postoperative phase but also over the longer term.

## DISCUSSION

This study investigated patient-reported outcomes at 12 months and whether early knee exercise within 4 h after TKA, using the HAL-SJ, improved knee function. The results showed that the KOOS QOL of the early HAL-SJ group showed significantly higher values than the control groups at 12 months. The recovery time for extension lag was significantly improved in the early HAL-SJ group compared with the HAL-SJ and control groups. Additionally, the knee flexion ROM in the early HAL-SJ group was significantly higher than those in the HAL-SJ and control groups at 1 week. To our knowledge, this is the first study to longitudinally evaluate the efficacy of HAL-SJ–assisted rehabilitation up to 12 months after TKA.

The early HAL-SJ group showed significantly better outcomes in terms of the KOOS QOL than the control groups at 12 months. TKA aims to improve symptoms and ADL, enhance health-related QOL, and prolong healthy life expectancy (12, 13). Hence, significant QOL score is the most important score for patients undergoing TKA. Previous studies reported that patient-reported outcomes significantly improved in the HAL-SJ group (12, 18, 20); however, the follow-up time was short. In addition, Collins et al. concluded that a minimal clinically important difference of at least 20 for all KOOS subscales represented an actual change in older patients who underwent TKA (23). The study showed significant differences in KOOS QOL between groups; however, only the early HAL-SJ group showed improvement, with a minimal clinically important difference of at least 20 on all KOOS subscales 12 months after TKA. Therefore, this study suggests early active-assistive support using the HAL-SJ can significantly improve long-term patient-reported outcomes.

The results showed that the early HAL-SJ group showed a significantly improved time needed for recovery of extension lag compared with the HAL-SJ and control groups. Extension lag has multiple causes, including pain due to surgery, knee swelling, and arthrogenic muscle inhibition of the quadricep (28). Previous studies have reported that HAL-SJ-based robotic-assisted rehabilitation results in reduced pain, improves ROM, and leads to an immediate improvement in extension lag compared with conventional rehabilitation (15, 16, 29–31). In addition, a meta-analysis reported that robotic exoskeletons significantly improved active and passive ROM and hospital stay length compared with conventional rehabilitation. (19). HAL-SJ detects BES generated by voluntary muscle contractions, even when movements are weak. This real-time feedback helps synchronize intended joint motion with robotic assistance, reinforcing the link between motor intention and execution. Recent studies have also addressed the clinical efficacy of the feedback effect of HAL therapy (32, 33). Furthermore, active-assistive movement with HAL-SJ may stimulate neuroplasticity by engaging motor pathways and enhancing corticospinal excitability, facilitating the reorganization of motor circuits crucial for recovery (20). Conversely, knee circumference and resting and walking NRS pain scores did not differ between the groups. Therefore, using the HAL-SJ within 4 h postoperatively suggests that the suppression of arthrogenic muscle inhibition and promotion of the central nervous system without exacerbation of swelling and pain in the knee might have contributed to the early improvement in the extension lag. However, the central nervous system mechanism of early improvement in the extension lag is unclear and requires further detailed studies, including those using electromyography.

The early HAL-SJ group had a better knee flexion ROM 1 week after surgery than the HAL-SJ and control groups. Previous studies have reported that early rehabilitation of knee joint mobilization after TKA improves knee ROM and gait pattern (4, 7, 8). The results of this study showed no significant effects for resting and walking pain among the group. Therefore, active-assisted mobilization using the HAL-SJ with knee flexion from 90° to 120° within 4 h after surgery likely contributed to the early improvements in flexion ROM. Oka et al. (34) reported that the knee flexion ROM at 5 days postoperatively indicated the likelihood of achieving the clinical goal of knee flexion ROM 12 months after TKA. In addition, early increased knee flexion resulted in significant restoration of a “normal” knee and functional improvement (35, 36). However, using the HAL-SJ improves the knee ROM in the early postoperative period but may not be effective based on long-term results. The reason for this could be that the frequency of HAL-SJ use was approximately 7 times in both groups, which might have been less frequent. Future studies should investigate the optimal frequency of use and the efficacy of long-term use.

### Study limitations

This study had some limitations. First, the primary outcome was a patient-reported outcome measure, and no blinding was used. As a result, participants’ awareness of their group allocation may have influenced their self-reported responses, introducing the possibility of information bias. Although patient-reported outcomes are essential for capturing perceived functional recovery after TKA, the lack of blinding should be considered when interpreting the results. Future studies incorporating blinding procedures, when feasible, or combining patient-reported outcomes with objective functional measures may help to further strengthen the validity of these findings. Second, this was a single-institution study in which all patients underwent a standardized surgical approach using the same implant. While this design ensured procedural consistency, it may have limited the representativeness of the study population. As a result, the generalizability of the findings to other clinical settings, patient populations, surgical techniques, or implant designs may be limited. Therefore, larger multi-institutional randomized controlled trials are warranted to confirm these findings. Third, we recognize that our study only assessed outcomes up to 12 months postoperatively. While we observed significant improvements in the early HAL-SJ group, long-term follow-up beyond 12 months is needed to evaluate whether these benefits are sustained over time. Finally, although our study focused on its effectiveness, we did not conduct a cost–benefit analysis, which limits the assessment of its practicality. Future research should evaluate whether functional improvements justify the financial burden, including both direct and indirect costs.

### Conclusions

Early HAL-SJ assisted rehabilitation was associated with improvements in KOOS QOL score at 12 months after TKA. Although improvements in knee flexion range of motion were observed in the early postoperative period, knee flexion and extension were secondary outcomes, and between-group differences were small, thus these findings should be interpreted with caution. Overall, the results suggest that HAL-SJ may be a feasible adjunct to early postoperative rehabilitation after TKA, although further studies are required to clarify its clinical relevance and long-term benefits.
